# Cellular ageing mechanisms in osteoarthritis

**DOI:** 10.1007/s00335-016-9641-z

**Published:** 2016-05-23

**Authors:** P. K. Sacitharan, T. L. Vincent

**Affiliations:** ARUK Centre for Osteoarthritis Pathogenesis, The Kennedy Institute of Rheumatology, University of Oxford, Oxford, England, UK

## Abstract

Age is the strongest independent risk factor for the development of osteoarthritis (OA) and for many years this was assumed to be due to repetitive microtrauma of the joint surface over time, the so-called ‘wear and tear’ arthritis. As our understanding of OA pathogenesis has become more refined, it has changed our appreciation of the role of ageing on disease. Cartilage breakdown in disease is not a passive process but one involving induction and activation of specific matrix-degrading enzymes; chondrocytes are exquisitely sensitive to changes in the mechanical, inflammatory and metabolic environment of the joint; cartilage is continuously adapting to these changes by altering its matrix. Ageing influences all of these processes. In this review, we will discuss how ageing affects tissue structure, joint use and the cellular metabolism. We describe what is known about pathways implicated in ageing in other model systems and discuss the potential value of targeting these pathways in OA.

Osteoarthritis (OA) is the most common form of arthritis worldwide and constitutes a huge societal burden (Woolf and Pfleger 2003; Glyn-Jones et al. 2015). This will likely increase as lifespan in the global population rises (Woolf and Pfleger 2003; Glyn-Jones et al. 2015). OA is a highly heterogeneous disease that affects all synovial joints, including the hand, knee, hip and spine and is characterised by the progressive degradation of the articular cartilage along with secondary bone remodelling and episodic synovitis (Vincent and Watt 2014). Ageing is the most important aetiological risk factor. Other important factors include obesity, genetics and, in younger individuals, acute destabilising joint injuries (Bijlsma et al. 2011). Several mechanisms have been proposed by which ageing impacts on the progression of joint degeneration in OA. This review will highlight and discuss the cellular metabolic mechanisms that are dysregulated in ageing cartilage and which may contribute to disease pathogenesis.

## Pathogenesis of OA

 Breakdown of the articular cartilage with remodelling of the underlying bone is the hallmark of OA. The articular cartilage is an exquisitely lubricated tissue located on the surface of the joint, responsible for smooth joint articulation (Pearle et al. 2005). Cartilage is avascular and aneural and contains just one cell type, the chondrocyte. 95 % of the cartilage volume is extracellular matrix, composed of predominantly type II collagen and the proteoglycan, aggrecan (Pearle et al. 2005). Chondrocytes are responsible for maintaining homeostatic cartilage turnover to renew and respond to changes in the mechanical environment, but excessive matrix catabolism can be driven by excessive mechanical joint loading, cytokines, growth factors and fragments of the extracellular matrix (ECM) (Wieland et al. 2005). Principle matrix-degrading enzymes include members of the ‘a disintegrin and metalloproteinase with thrombospondin motif’ (ADAMTS) family (largely ADAMTS5) and the matrix metalloproteinase (MMP) family (largely MMP1, 8 and 13), which degrade aggrecan and collagen, respectively (Nagase et al. 2006). The significance of these enzymes in vivo was demonstrated by showing that mice deficient in either ADAMTS5 or MMP13 had reduced cartilage degradation scores following surgically induced murine OA (Glasson et al. 2005; Little and Smith 2008). Interleukin 1-beta (IL-1β) and tumour necrosis factor-alpha (TNFα) are capable of inducing and activating these catabolic enzymes to degrade cartilage (Saklatvala 1981, 1986). However, there is scant evidence that these cytokines are central in driving disease in vivo (Clements et al. 2003; Glasson 2007; Fukai et al. 2012).These enzymes can be induced rapidly upon surgical joint destabilisation in a highly mechano-sensitive manner as well; gene regulation and disease is abrogated if the joint is immobilised following surgery. This suggests that mechanical factors can also initiate pathogenic pathways (Burleigh et al. 2012). This accords well with epidemiological evidence that OA may principally be driven by mechanical joint overload and injury (Brandt et al. 2009; Nagase et al. 2006; Nagase and Kashiwagi 2003).

 The significance of joint inflammation in contributing to tissue breakdown is unclear. Infiltration of mononuclear cells into the synovial membrane is observed in human OA (Scanzello and Goldring 2012) and there is much evidence to support activation of the innate immune system in disease (Orlowsky and Kraus 2015). It seems likely that inflammation, when present, will exacerbate tissue breakdown and contribute to painful episodes of disease.

## Age and osteoarthritis

**Fig. 1:**
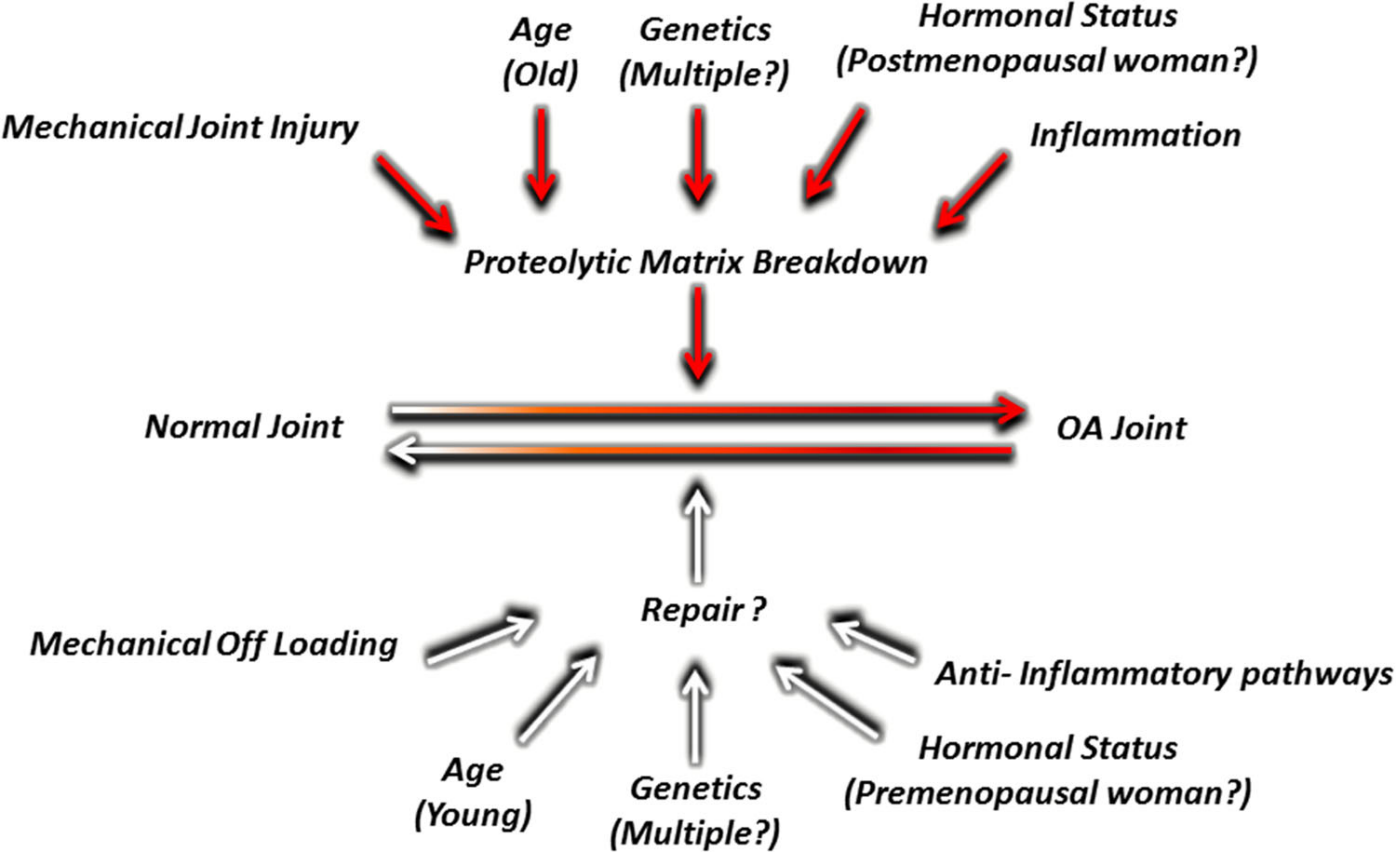
OA pathogenesis. Proteolytic matrix breakdown is a key feature of cartilage breakdown in OA. This is influenced by risk factors such as age, inflammation and mechanical injury. The ability of cartilage to repair is controversial but is also likely to be influenced by similar classes of modulators that may hinder or aid repair

The mechanisms by which age affects cartilage health are multiple and varied but probably not simply due to accumulated ‘wear and tear’ over time (Fig. 1). It is well established that type II collagen has negligible turnover during the normal adult lifetime but becomes biochemically modified (crosslinked) with age (Bank et al. 1998). Cartilage proteoglycan, whilst having a much shorter half-life, is also modified with age (Hickery et al. 2003; Lauder et al. 2001). Collectively, this significantly changes the stiffness of the matrix (Bank et al. 1998) and likely affects not only its ‘brittleness’, making it more susceptible to fracture, but also its ability to sense and respond to mechanical load. Indeed, the impaired ability of aged cartilage to activate transforming growth factor beta 1 (TGFβ1)-dependent signals upon mechanical compression may be related to this (Madej et al. 2015). Interestingly, Loeser et al. (2013) used a surgical OA model in 12-week-old and 12-month-old male mice. Not only did the older mice exhibit increased OA disease scores, but gene expression profiles were also significantly different in the aged mice (Loeser et al. 2013).

Mechano-protective mechanisms are also substantially diminished in ageing. Such mechanisms include protection afforded by muscle ‘splinting’ of the joint upon weight-bearing activity. Muscle atrophy is highly prevalent in elderly populations (Brook et al. 2016) and very likely contributes to loss of joint protection during the normal gait cycle with age. This is amplified by loss of gait reflexes whereby deceleration of movement upon heel strike would normally occur in a younger individual but is lost in the elderly (reviewed in Brandt et al. 2009).

**Fig. 2:**
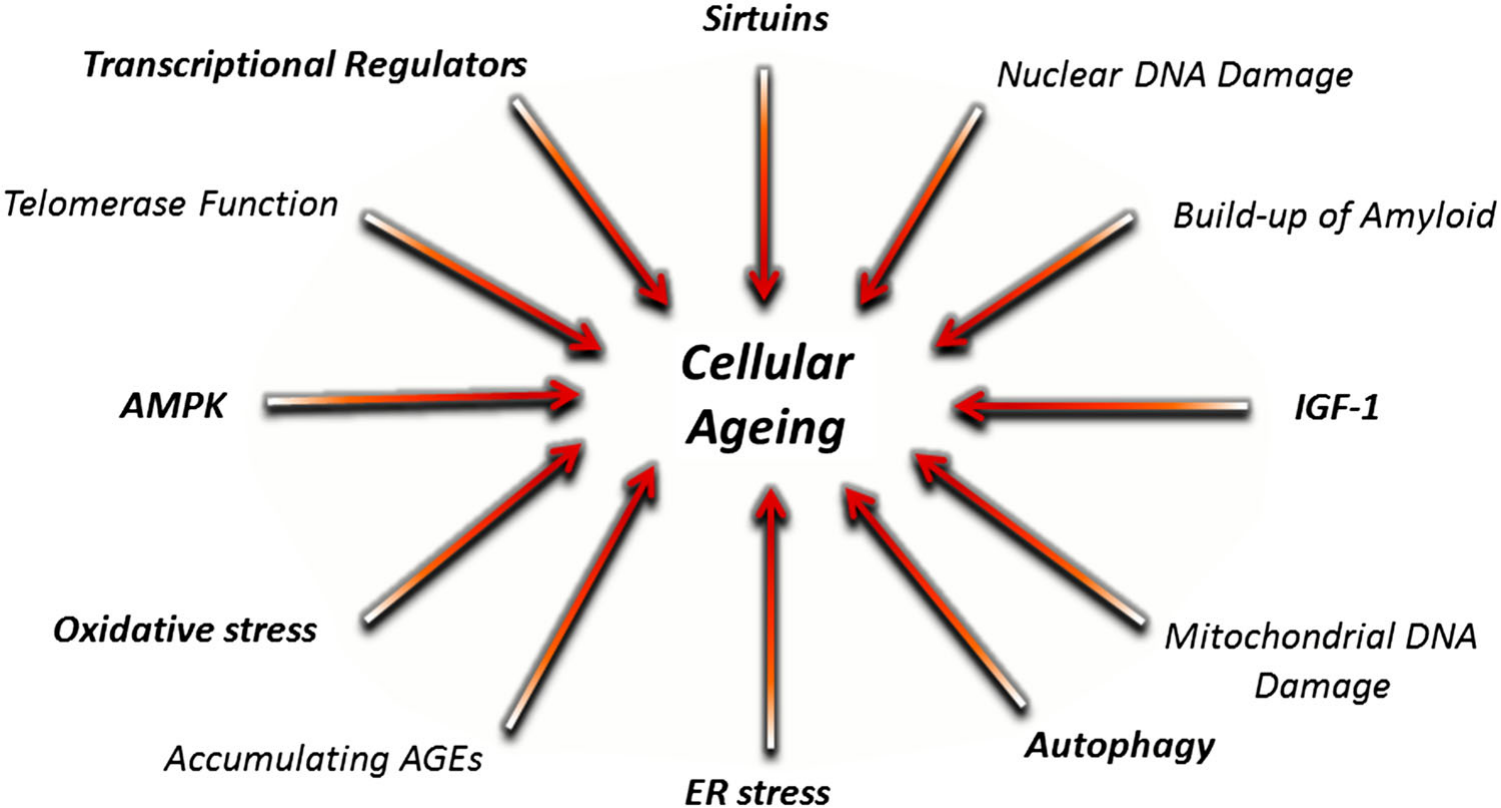
Cellular ageing mechanisms. Many cellular signalling mechanisms have been implicated in the regulation of ageing in mammals. This review will discuss the metabolic cellular mechanisms (in bold) described to be important in chondrocytes, cartilage and the OA joint

One significant advance over the past decade or so has been the characterisation of the cellular metabolic pathways that are affected by ageing (Fig. 2). Largely, these pathways have been defined in other model systems and in recent years studied in the joint. Despite global recognition that OA is a whole joint disease, most studies to date have focused on cartilage when studying ageing mechanisms in OA. The rest of this review will focus on how ageing pathways are affected in joint cells and the likely significance of these pathway changes on cartilage degradation in OA.

## Autophagy

Autophagy is a cellular homeostatic mechanism for the removal of dysfunctional organelles and proteins (Boya et al. 2013). Defective autophagy is a key feature of age-related diseases and recent observations indicate that this process is compromised in ageing cartilage (Rubinsztein et al. 2011; Lotz and Caramés 2011). Caramés et al. (2010) examined key autophagy proteins in human and murine cartilage. These markers were Unc-51-like kinase 1 (ULK1), an inducer of autophagy, Beclin1, a regulator of autophagy, and microtubule-associated protein 1 light chain 3 (LC3), which executes autophagy (Lotz and Caramés 2011). The expression of ULK1, Beclin1 and LC3 protein was reduced in human OA chondrocytes and cartilage (Caramés et al. 2010). In mouse knee joints, decreased protein expression of ULK1, Beclin1 and LC3 was associated with proteoglycan loss and increased apoptosis at 9 and 12 months of age and following induction of OA by joint destabilisation (Caramés et al. 2010). A further study by Caramés et al. (2015) showed that 28-month-old mice had significant reduction in the total number of autophagocytic vesicles per cell in articular cartilage compared with young 6-month-old mice. Interestingly, Bouderlique et al. (2015) generated a cartilage-specific inducible ATG5 KO (ATG5cKO) mouse which developed severe OA with increased cell death when aged. Surprisingly, no difference in the development of post-traumatic OA was observed between ATG5cKO and control mice post DMM (Bouderlique et al. 2015).

Studies have also examined the role of mammalian target of rapamycin (mTOR) signalling which when activated inhibits ULK1 and in turn inhibits autophagy. Zhang et al. (2014) reported upregulation of mTOR expression that correlated with increased chondrocyte apoptosis and reduced expression of key autophagy genes in human OA tissue. In addition, inducible cartilage-specific mTOR KO mice displayed increased autophagy signalling and significant protection from surgically induced OA associated with a significant reduction in the apoptosis (Zhang et al. 2014). Carames et al. (2012) used rapamycin to pharmacologically inhibit mTOR in mice. The severity of cartilage degradation was significantly reduced in the rapamycin-treated group compared with the control group and this was associated with a significant decrease in synovitis 10 weeks post-surgery (Carames et al. 2012). Rapamycin treatment also maintained cartilage cellularity and decreased ADAMTS5 and IL-1β expression in articular cartilage (Carames et al. 2012).

## Sirtuins

The well-conserved Sirtuin gene family has been strongly associated with longevity since silent information regulator 2 was shown to extend lifespan in budding yeast, worms, flies and mice (Giblin et al. 2014). In mammals, Sirtuins are a seven-member family (SirT1-7) of NAD-dependent deacetylases, with SirT4 and SirT6 also demonstrating ADP-ribosyltransferase activity (Verdin 2014). The Sirtuins are involved in a wide range of physiological systems and cellular functions including cell metabolism, apoptosis, growth, development, inflammation and stress responses (Sacitharan et al. 2012; Morris 2013). To date, only SirT1 and SirT6 have been investigated in OA.

Mice with SirT6 haploinsufficiency fed on high-fat diet had increased OA scores at 6 months of age compared with wild-type animals fed on a similar diet (Ailixiding et al. 2015). No change in OA score was reported between mice with SirT6 haploinsufficiency and control mice fed on a normal diet (Ailixiding et al. 2015). Wu et al. (2015) reported significantly decreased SirT6 protein expression levels in articular chondrocytes of OA patients compared with normal human chondrocytes. In addition, intraarticular injection of lentiviral-driven SirT6 in mice reduced OA disease scores 8 weeks post-surgical destabilisation compared with mice receiving the lentiviral vector control (Wu et al. 2015).

Most Sirtuin-focussed OA research to date has been around SirT1. SirT1 expression is decreased in human articular chondrocytes in response to nutritional, catabolic or mechanical stress (Takayama et al. 2009). Dvir-Ginzberg et al. (2008) showed that overexpression of SirT1 in chondrocytes isolated from articular cartilage of human joints resulted in an increase in cartilage-specific gene expression of collagen 2(α1) (COL2A1) through the deacetylation of SOX-9 (Dvir-Ginzberg et al. 2008). Similarly, decreased expression of aggrecan, COL2A1 and collagen 9(α1) was observed in SirT1 siRNA-treated human knee chondrocytes (Fujita et al. 2011). Conversely, decreasing SirT1 resulted in the upregulation of collagen 10(α1) and ADAMTS5 gene expression (Fujita et al. 2011).

Gabay et al. (2012) demonstrated that SirT1 constitutive whole-body knockout mice had increased OA disease scores and exhibited low levels of COL2A1, ACAN, GAG release and high protein levels of MMP-13. These studies were performed at 3 weeks of age as constitutive SirT1 KO mice only survive for a few weeks post-partum. The same group generated mice in which a point mutation renders SirT1 inactive, had increased levels of apoptotic chondrocytes and increased OA disease scores when compared with age-matched (9 months) wild-type control mice (Gabay et al. 2013). Matsuzaki et al. (2013) generated cartilage-specific SirT1 conditional knockout (Sirt1-cKO) mice using an inducible collagen II-driven Cre recombinase (COL2A1-ERT2 Cre). Sirt1-cKO mice showed accelerated OA progression at 2 and 4 (but not surprisingly 8) weeks post joint destabilisation. In addition, spontaneous OA scores were significantly higher in 1-year-old Sirt1-cKO mice than in control mice (Matsuzaki et al. 2013). Together, these studies to date suggest that SirT1 may have a protective role in cartilage. The specific targets of SirT1 and precise mechanisms of action are as yet unclear.

## IGF1

The evolutionarily conserved insulin/IGF1 signalling (IIS) pathway plays a key role in hormonal regulation during ageing (van Heemst 2010). IGF1 has potent anabolic effects on cells and downstream activation of cell proliferation, modulation of tissue differentiation and protection from apoptosis (van Heemst 2010). Mice with mutations in the IGF1 receptor display altered longevity. Female but not male mice with a heterozygous IGF1R mutation lived longer and demonstrated increased resistance to oxidative stress (Holzenberger et al. 2003). In addition, Klotho, a transmembrane protein which when overexpressed inhibits IGF1 signalling in mice extends lifespan (Kurosu et al. 2005). Klotho mutant mice age prematurely (Kuro-o 2009).

The role of IGF1 in articular cartilage metabolism has been extensively investigated as well. Interestingly, IGF1 was identified by Salmon and Daughaday (1957) and originally named as “sulphation factor” because of its ability to stimulate 35-sulphate incorporation into rat cartilage. Exogenous IGF1 when added to monolayers of bovine chondrocytes or cartilage explants increases proteoglycan synthesis (Sah et al. 1994). Furthermore, a combination of IGF1 and TGFβ1 has been shown to regulate proliferation and differentiation of periosteal mesenchymal cells during chondrogenesis (Fukumoto et al. 2003). However, addition of IGF1 alone did not have the same effect on chondrogenesis (Fukumoto et al. 2003). This result validates the experiments conducted by Tsukazaki et al. (1994) who showed the ability of TGFβ1 to increase the number of IGF1 receptors in chondrocytes without changing their affinity. IGF1-deficient rats developed articular cartilage lesions (Ekenstedt et al. 2006) and in murine and equine models addition of IGF1 to chondrocyte grafts enhanced chondrogenesis in cartilage defects (Fortier et al. 2002; Goodrich et al. 2007). These studies further support the need for IGF1 to maintain articular cartilage integrity. Several studies have demonstrated that the ability of chondrocytes to respond to IGF1 decreases with age and in OA (Loeser et al. 2000; Morales 2008).

## Oxidative stress

Reactive oxygen species (ROS) are produced as a result of cellular metabolism and environmental factors (Nathan and Cunningham-Bussel 2013). ROS can damage nucleic acids and proteins, thus altering their functions (Gorrini et al. 2013). Hence, cells produce antioxidants to counteract oxidative stress caused by ROS (Gorrini et al. 2013; Nathan and Cunningham-Bussel 2013). Accumulation of nitrotyrosine, a marker of protein oxidation, is seen in ageing normal human and monkey cartilage and OA human cartilage (Loeser et al. 2002). Oxidative stress with ageing has been shown to make human chondrocytes more susceptible to apoptosis through the dysregulation of the glutathione antioxidant system (Carlo and Loeser 2003). Reduced levels of catalase were observed in aged rat chondrocytes (15–18 months) compared with chondrocytes from young adult rats (6 months old) (Jallali et al. 2005). Furthermore, reductions in mitochondrial superoxide dismutase, manganese-superoxide dismutase and glutathione peroxidase were all observed in isolated chondrocytes from OA tissue samples (Aigner et al. 2006; Ruiz-Romero et al. 2006, 2009). ROS are also produced by chondrocytes in response to stimulation by cytokines and growth factors, including IL-1, TNFα and TGFβ1 (Lo and Cruz 1995; Jallali et al. 2007; Loeser 2012). Baker et al. (1988) demonstrated that hydrogen peroxide (H2O2) suppressed proteoglycan synthesis in human cartilage explant culture. This could be potentiated when catalase and the glutathione peroxidase/reductase systems were inhibited (Baker et al. 1988). The role of oxidative stress mediators in cartilage health is unknown although it is interesting that H2O2 is an important tissue injury signal in other model systems and drives repair responses (Razzell et al. 2013).

## ER stress and UPR

The ER is arranged in a dynamic tubular network and protein maturation at the ER is vital for the correct folding of proteins (Hetz 2012). Several feedback mechanisms are in place to cope with impaired protein folding which causes ER stress and may lead to apoptosis (Hetz 2012). These coping mechanisms together are known as the unfolded protein response (UPR) (Ron and Walter 2007). The UPR signalling network is complex and requires several key components (Ron and Walter 2007). UPR stress sensors, inositol-requiring protein 1α (IRE1α), protein kinase RNA-like endoplasmic reticulum kinase (PERK) and activating transcription factor 6 (ATF6), signal information about the folding status of the ER proteins to the cytosol and nucleus to suppress new protein synthesis and restore optimal protein-folding capacity (Ron and Walter 2007; Hetz 2012). The transcription factors mentioned above activate UPR target genes involved in pathways such as autophagy, apoptosis, lipid synthesis and NF-κB signalling thereby minimising the effects of ER stress (Ron and Walter 2007; Hetz 2012).

In vitro studies have shown increased UPR in OA articular chondrocytes predominately through the PERK and IRE1 pathways (Liu-Bryan and Terkeltaub 2015). In addition, Husa et al. (2013) showed that biomechanical injury, IL-1β and nitric oxide (NO) increase ER stress and the UPR in cultured bovine chondrocytes. Interestingly, transfection of CHOP in ‘gain of function’ experiments sensitised normal human chondrocytes to IL-1β-induced NO and MMP3 release without inducing these responses by itself (Husa et al. 2013). It must be noted that excessive CHOP signalling induces ER stress as well, by increasing protein synthesis (Ron and Walter 2007; Hetz 2012). Hence CHOP signalling needs to be finely regulated. Surgically induced OA in mice in which CHOP was constitutively deleted were partially protected from increased chondrocyte apoptosis and cartilage degradation (Uehara et al. 2014), although there was no difference in ER stress between CHOP KO mice and control mice (Uehara et al. 2014).

XBP1, a UPR transcription factor, has a role in chondrocyte differentiation. Overexpression of XBP1 in chondrocyte cell lines led to accelerated chondrocyte hypertrophy as shown by increased expression of type X collagen and Runt-related transcription factor 2 (RUNX2) (Liu et al. 2012). Conversely, knockdown of XBP1 by siRNA abolished hypertrophic chondrocyte differentiation (Liu et al. 2012). Takada et al. (2011) showed enhanced apoptosis in XBP1-induced ATF6 signalling in osteoarthritic cartilage. As several new compounds for alleviating ER stress are being developed and tested in other disease areas (Hetz 2012), it is likely that studies will soon follow in OA models.

## AMPK

The serine/threonine kinase AMP-activated protein kinase (AMPK) is a master regulator of cellular energy and adjusts to changes in energy demand (Salminen and Kaarniranta 2012). Terkeltaub et al. (2011) first described normal human knee articular chondrocytes which expressed AMPKα1, α2, β1, β2 and γ1 subunits with constitutive and robust activity. However, AMPK activity was decreased in OA articular chondrocytes and cartilage and in normal chondrocytes treated with IL-1β and TNFα (Terkeltaub et al. 2011). Knockdown of AMPKα resulted in enhanced catabolic responses to IL-1β and TNFα in chondrocytes (Terkeltaub et al. 2011). AMPK activators (AICAR and A-769662) suppressed pro-catabolic responses to IL-1β and TNFα from chondrocytes (Terkeltaub et al. 2011). A further study by the same group revealed that AMPK activity was decreased in mouse knees post-surgical destabilisation and in aged knee cartilage (6–24 months), as well as in bovine chondrocytes after biomechanical injury (Petursson et al. 2013). This study further identified an upstream kinase, the liver protein kinase B1 (LKB1) as the promoter of AMPK activity in chondrocytes (Petursson et al. 2013). Knockdown of LKB1 attenuated chondrocyte AMPK activity and increased NO, MMP3 and MMP13 release in response to IL-1β and TNFα (Petursson et al. 2013). LKB1, like AMPK, also decreased in diseased and aged murine knee cartilage (Petursson et al. 2013). In addition, pre-treatment of bovine chondrocytes with AMPK activators inhibited the catabolic response of NO after biomechanical injury (Petursson et al. 2013).

## Other transcriptional regulators

FOXO transcription factors are involved in the regulation of the cell cycle, apoptosis, metabolism and autophagy (Eijkelenboom and Burgering 2013). Multiple and diverse upstream pathways regulate FOXO activity through post-translational modifications and nuclear-cytoplasmic shuttling (Eijkelenboom and Burgering 2013). Healthy human cartilage (from humans aged between 23 and 90) expresses FOXO1 and FOXO3 but not FOXO4 protein subtypes (Akasaki et al. 2014a, b). During ageing, expression of FOXO1 and FOXO3 markedly decreases in the superficial zone of human cartilage regions exposed to maximal weight bearing (Akasaki et al. 2014a, b). Similar patterns of FOXO expression have been observed in mice upon ageing (4–24 months) and following joint destabilisation (Akasaki et al. 2014a, b). FOXO1 protein expression was suppressed in human chondrocytes when cultured with IL-1β and TNFα, while TGFβ increased FOXO1 and FOXO3 protein expression (Akasaki et al. 2014a, b). Akasaki et al. 2014a, b reported reduced expression of FOXO transcription factors and increased cell death following oxidative stress in chondrocytes.

Interestingly, this increase in cell death was accompanied by reduced levels of antioxidant proteins (glutathione peroxidase 1 and catalase) and autophagy-related proteins (LC3 and Beclin1) (Akasaki et al. 2014a, b). These studies point to a tissue-specific signature of FOXO expression and its partial role in regulating oxidative stress resistance and autophagy. In vivo studies using FOXO cartilage-specific KO mice or FOXO activators and inhibitors have not yet been reported.

Another family of transcription factors, known as peroxisome proliferator-activated receptors (PPARs), have been shown in recent studies to be involved in OA. PPARs are ligand-activated transcription factors that are involved in regulating glucose and lipid homeostasis, inflammation, proliferation and differentiation (Peters et al. 2012). Three PPAR isoforms, PPARα, PPARβ/δ (also known as PPARβ or PPARδ) and PPARγ, are found in all mammals (Peters et al. 2012). It has been known for some time that PPARγ inhibits IL-1β-induced proteoglycan degradation (Francois et al. 2006). Vasheghani et al. (2013) reported that (constitutive) cartilage-specific disruption of PPARγ results in spontaneous OA in mice (14 months of age). The same group went on to generate an inducible cartilage-specific PPARγ KO mouse (PPARγ-cKO) (Vasheghani et al. 2015). Postnatal deletion of PPARγ in chondrocytes upon administration of doxycycline did not lead to spontaneous OA, but the mice became more susceptible to experimental OA (Vasheghani et al. 2015). PPARγ-cKO mice displayed increased expression of MMP13 and ADAMTS5, and had higher numbers of apoptotic chondrocytes in OA knee joints (Vasheghani et al. 2015). This same group previously reported that mTOR KO mice were protected from experimental OA by increasing autophagy (Zhang et al. 2014). Therefore, the group tested the hypothesis that accelerated OA in PPARγ-cKO mice was due to enhanced mTOR signalling. PPARγ-cKO had increased expression of mTOR and a decrease in autophagy markers in naive and destabilised joints (Vasheghani et al. 2015). Double-KO mice of mTOR and PPARγ were protected from surgically induced OA with a phenotype similar to that of the previously published mTOR KO (Zhang et al. 2014; Vasheghani et al. 2015). These studies support a role for PPARγ in mTOR-driven chondroprotection. Consistent with this, Pioglitazone (a PPARγ agonist) has been shown to reduce cartilage lesions in an experimental dog model of OA (Boileau et al. 2007). Studies to date make PPARγ an attractive future drug target for OA.

Studies have also been conducted to elucidate the role of PPARα and PPARβ/δ in OA. Clockaerts et al. (2011) reported that the PPARα agonist Wy-14643 inhibited mRNA expression of MMP1, MMP3 and MMP13 in human OA cartilage explants after IL-1β treatment but did not have an effect on COL2A1 or aggrecan expression. This study suggests a possible protective effect of PPARα in OA also. In contrast, Ratneswaran et al. (2015) looked at the role of PPARβ/δ in OA. PPARβ/δ activation by GW501516 increased expression of several proteases (MMP2, MMP3 and ADAMTS2) in murine chondrocytes and increased aggrecan degradation and GAG release in knee joint explants. Constitutive cartilage-specific PPARβ/δ KO displayed no developmental phenotype and showed marked protection in the surgical destabilisation model of OA (Ratneswaran et al. 2015). This study suggests PPARβ/δ to have a possible protective role in OA. The role of each PPAR isomer seems to be very distinct. Hence careful targeting of these transcription factors might be required if one is to envisage them being potential therapeutic targets in OA.

## Conclusions and future directions

**Fig. 3:**
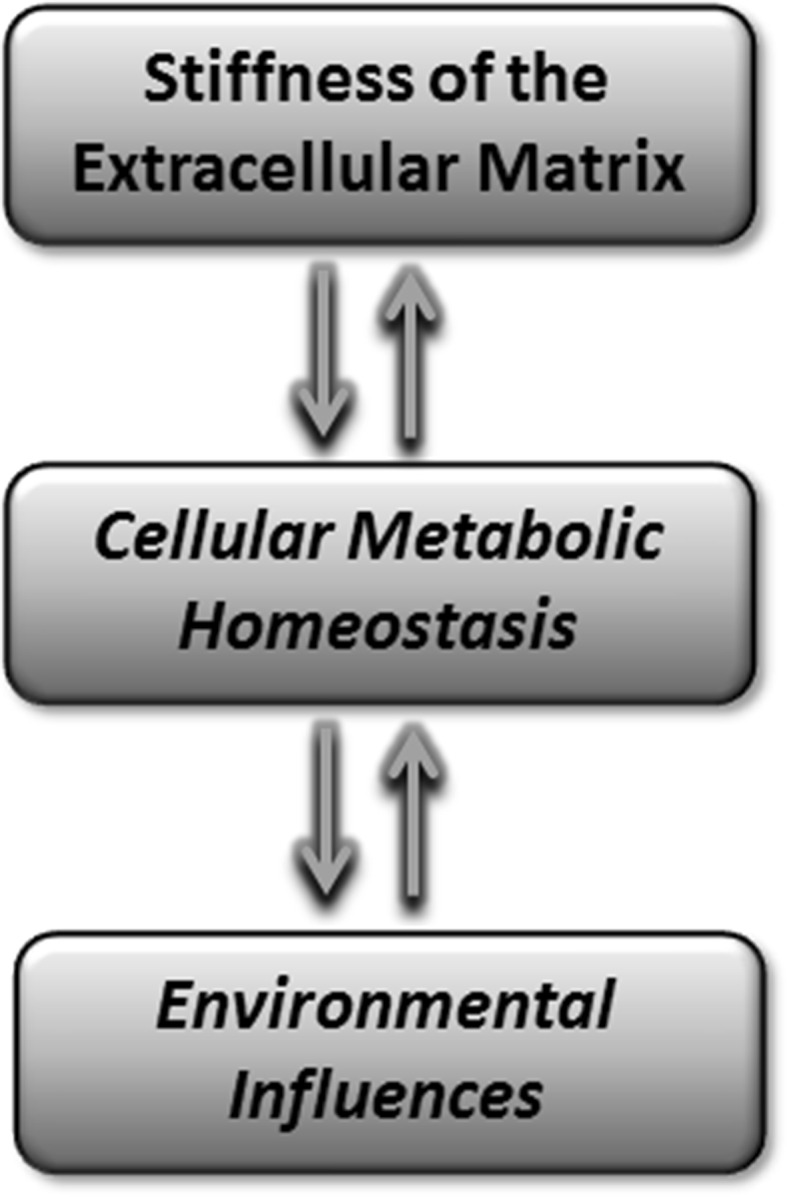
Ageing processes affecting joint health. Age-related changes to the extracellular matrix result in increased stiffness, which changes the mechano-responsiveness of chondrocytes and brittleness of the tissue. Environmental influences include neuromuscular decline, changing hormonal status, activity levels and diet. There is a dynamic interplay between these influences and cellular metabolic mechanisms in chondrocytes to regulate cartilage ageing

Recent advances in basic science have improved our understanding of how cellular ageing may contribute to the pathogenesis of OA. We now better appreciate the complex signalling networks that control metabolic processes and regulators in articular chondrocytes. The dysregulation of these factors may change cellular responses to inflammatory and anabolic signals as well as affect the mechano-responsiveness of the tissue (Fig. 3). How these pathways are regulated in other joint tissues and the impact on the intrinsic and extrinsic repair responses are unknown. Pragmatic approaches to disease management will need to include how changes in body weight, nutrition, exercise, comorbidities and risk factors influence these cellular responses to ageing in different OA patient cohorts (Fig. 3). Specific pharmacological approaches targeted at some of these pathways may be a realistic future vision.
